# Effect of Light Acclimation on the Organization of Photosystem II Super- and Sub-Complexes in *Arabidopsis thaliana*

**DOI:** 10.3389/fpls.2016.00105

**Published:** 2016-02-17

**Authors:** Ludwik W. Bielczynski, Gert Schansker, Roberta Croce

**Affiliations:** Biophysics of Photosynthesis/Energy, Faculty of Sciences, Department of Physics and Astronomy, VU University AmsterdamAmsterdam, Netherlands

**Keywords:** *Arabidopsis thaliana*, light intensity acclimation, BN-PAGE, 2D-PAGE, PSII

## Abstract

To survive under highly variable environmental conditions, higher plants have acquired a large variety of acclimation responses. Different strategies are used to cope with changes in light intensity with the common goal of modulating the functional antenna size of Photosystem II (PSII). Here we use a combination of biochemical and biophysical methods to study these changes in response to acclimation to high light (HL). After 2 h of exposure, a decrease in the amount of the large PSII supercomplexes is observed indicating that plants are already acclimating to HL at this stage. It is also shown that in HL the relative amount of antenna proteins decreases but this decrease is far less than the observed decrease of the functional antenna size, suggesting that part of the antenna present in the membranes in HL does not transfer energy efficiently to the reaction center. Finally, we observed LHCII monomers in all conditions. As the solubilization conditions used do not lead to monomerization of purified LHCII trimers, we should conclude that a population of LHCII monomers exists in the membrane. The relative amount of LHCII monomers strongly increases in plants acclimated to HL, while no changes in the trimer to monomer ratio are observed upon short exposure to stress.

## Introduction

Land plants appeared on Earth around 568–815 million years ago (Clarke et al., 2011). On an evolutionary time scale, this is enough time to evolve highly sophisticated acclimation responses to allow survival as a sessile organism under variable environmental conditions. As photoautotrophs, the acclimation responses to different light conditions are essential adaptations. They provide balance between the harvesting of enough energy for metabolic and anabolic processes and the protection against excess excitation energy (EEE). EEE represents a risk associated with the light-harvesting systems as it increases the probability of reactive oxygen species (ROS) generation, which can be highly destructive for the photosynthetic apparatus and the cell (reviewed in Asada, 2006).

As light interception occurs in the chloroplast, it is there that the first steps and coordination of the light intensity acclimation take place. From all the complexes involved in the light phase of photosynthesis, Photosystem II (PSII) is the major target of acclimation. Its composition, functionality and amount are dynamically adjusted in response to changes in light conditions (Ballottari et al., 2007; Betterle et al., 2009; Johnson et al., 2011; Belgio et al., 2014; Kono and Terashima, 2014; Dietz, 2015; Suorsa et al., 2015).

Upon exposure to HL, short term responses are activated in a range from seconds to minutes. This time is only sufficient for rearrangement of the chloroplast components, without an influence of biosynthesis or degradation. Protonation and phosphorylation of different components (Allen, 1992; Fristedt and Vener, 2011; Wientjes et al., 2013a,b; Pietrzykowska et al., 2014) trigger the processes known as non-photochemical quenching (NPQ; Ruban et al., 2012) and state transitions (Tikkanen and Aro, 2012).

Long-term acclimation occurs in a range from hours to weeks and involves selective synthesis and degradation of chloroplast components. It also involves phosphorylation of some of the components (Fristedt and Vener, 2011). As a consequence of PSII antenna size adjustments, the chlorophyll (Chl) *a*/*b* ratio decreases in increasing light intensities (Park et al., 1997; Ballottari et al., 2007; Wientjes et al., 2013c) and the density of PSII supercomplexes in the thylakoid membrane is modified (Kouřil et al., 2013).

The current model of PSII comes from the crystal structure of PSII from the cyanobacterium *Thermosynechococcus vulcanus* (Umena et al., 2011). In combination with other methods, up to 40 protein subunits that compose PSII were identified (reviewed Shi et al., 2012). More than half have a molecular mass below 15 kDa and are expressed under specific environmental conditions (Plöscher et al., 2009). The reaction center (RC) complex is a heterodimer composed of the products of the genes PsbA (D1) and PsbD (D2), binding in total six Chl *a* and two pheophytins (Umena et al., 2011). Associated to the RC are the internal antennae CP47 (PsbB) and CP43 (PsbC) binding 16 and 13 Chl *a*, respectively, and a number of small subunits (Shi et al., 2012). In higher plants this complex is called the PSII core (C).

In plants the core is supplemented with an outer antenna system, composed of Chl *a*/*b* binding proteins known as light-harvesting complexes (LHC), forming supercomplexes (Dekker and Boekema, 2005). PSII supercomplexes exist in different configurations, containing a variable number of LHCIIs (heterotrimers of Lhcb1-3) and minor antennae (Caffarri et al., 2009). LHCII has two possible docking sites on the core, where it can bind with different affinities: strongly (S) through CP43 and CP26 (Lhcb5), and with moderate affinity (M) on the CP47 side through CP24 (Lhcb6) and CP29 (Lhcb4). Most of the PSII complexes are in dimeric form (C_2_) and can bind several LHCIIs, forming C_2_S, C_2_M, C_2_S_2_, C_2_SM, C_2_S_2_M, and C_2_S_2_M_2_ supercomplexes. In addition two complexes containing monomeric core were observed: the naked core (C) and the core with a strongly bound LHCII (CS).

Photosystem II heterogeneity is partially a result of the repair cycle of the D1 protein (Aro et al., 2005). Photodamage of PSII due to radicals and oxygen species formation is an intrinsic property of PSII. As a consequence, plants constantly replace PSII in the light. The process is multiphasic involving (i) phosphorylation of different subunits, (ii) monomerization and migration to the stroma lamellae, (iii) partial disassembly of PSII core, (iv) proteolysis of damaged proteins, (v) replacement of the damaged D1 protein, and (vi) reassembly, dimerization and photoactivation of PSII.

Besides the structural and functional heterogeneity due to the balance between photoinhibition and repair cycle, the PSII supercomplexes are rearranged during light acclimation (Ballottari et al., 2007; Kouřil et al., 2013). The adjustment is achieved through regulation of the expression levels of Lhcb1-3 and Lhcb6 proteins. In plants, most of the PSII supercomplexes are randomly distributed surrounded by an extra pool of LHCII loosely associated with them (called “extra” LHCII). In HL the decrease in the antenna size mainly affects the size of this extra LHCII pool (Wientjes et al., 2013c) leading to a denser PSII packing (Kouřil et al., 2013). The PSII supercomplexes in some parts of the grana are also organized in semicrystaline arrays. The most common semicrystaline structure under all light conditions is composed of C_2_S_2_M_2_ supercomplexes. However, in the thylakoids from HL acclimated plants, due to the decrease of M trimers, some C_2_S_2_ semi-crystalline structures were also observed (Kouřil et al., 2013).

Upon a short light stress treatment, plants switch on a series of mechanisms known as NPQ. The main NPQ component (qE) reflects a process by which EEE is dissipated as heat. The process is triggered by low lumenal pH that activates the PsbS protein (Li et al., 2000) and the xanthophyll cycle (Demmig-Adams et al., 1990; reviewed in Jahns and Holzwarth, 2012). It was suggested that the pH dependent NPQ is induced by allosteric conformational changes and aggregation of peripheral LHCII (Horton and Ruban, 2005; Horton et al., 2008). Changes in the PSII antenna size were also suggested based on biochemical (Betterle et al., 2009), and functional (Holzwarth et al., 2009) data and supported by structural evidence (Johnson et al., 2011). However, more recently it was proposed that the effective antenna size of PSII is even increasing during NPQ (Belgio et al., 2014).

In this work, we have studied the structural changes of PSII during short HL stress and long-term acclimation to different light intensities by combining quantitative biochemical analysis with functional measurements performed on the same plants. This has allowed us to get a more complete picture of the effect of light acclimation on the composition and functional organization of the photosynthetic complexes.

## Materials and Methods

### Plant Material

*Arabidopsis thaliana* (ecotype Col-0) WT seeds were sown on Murashige and Skoog (MS) medium agar plates. After 5–7 days the seedlings were transplanted to final pots. Plants were grown for 7 weeks in growth chambers (AR-36L, Plant Climatics Percival) at 70% RH, 21°C, a photoperiod of 8/16 h (day/night) and under 200 or 600 μmol photons^∗^m^–2∗^s^–1^. After 3 weeks, a batch of plants grown under 200 μmol photons^∗^m^–2∗^s^–1^ was transferred and grown for an additional 3 weeks at 1800 μmol photons^∗^m^–2∗^s^–1^ (FytoScope FS 3400, Photon Systems Instruments). For the short HL stress experiment the plants were grown as previously described under 200 μmol photons^∗^m^–2∗^s^–1^ and then after 6 weeks of growth, transferred for 0.5, 2 and 6 h to 1800 μmol photons^∗^m^–2∗^s^–1^. Plants illuminated with growth light (200 μmol photons^∗^m^–2∗^s^–1^) for 6 h were used as a control.

### Thylakoid Isolation

If not stated otherwise the plants were harvested after a night in darkness. The plants from short-term HL stress were harvested and immediately transferred to an ice bath, where they stayed until thylakoid isolation. The isolation procedure was described in Robinson et al. (1980), and modified according to Caffarri et al. (2009). Isolated thylakoid membranes were resuspended in the storage buffer (20 mM HEPES, pH 7.5, 0.4 M sorbitol, 15 mM NaCl and 5 mM MgCl_2_). The samples were rapidly frozen in liquid nitrogen and stored at –80°C.

### Pigment Isolation

The amount of chlorophylls on a leaf fresh weight basis, Chl *a*/*b* ratio and chlorophyll/carotenoid (Chl/Car) ratio were determined from absorption spectra of 80% acetone extracts measured with a Carry 4000 spectrophotometer (Varian). The absorption spectra were fitted with the spectra of individual pigments in the same solvent, as described in Croce et al. (2002). The quantification of different carotenoids was performed by HPLC using a System Gold 126 Solvent module and 168 Detector (Beckman Coulter) as described by Gilmore and Yamamoto (1991) with the modification reported in Xu et al. (2015).

### 2D-PAGE Analysis

For thylakoid membrane complex quantification a BN-PAGE was performed in a gel (4% stacking and 4–12.5% resolving gel) polymerized from a bisacrylamide/acrylamide mixture with a ratio of 32:1 (Järvi et al., 2011). The gels were cast from the bottom, in batch to decrease the mixing of the top layer using a Mini-PROTEAN 3 Multi-Casting Chamber (Bio-Rad). To prevent the mixing of butanol (used to get a straight gel top) with the top layers of the resolving gel the glycerol gradient in the gel was modified from 0–20% to 5–20% and a cushion of water was put on the top before casting. The gels were left overnight at room temperature (RT) to assure good polymerization of the low-acrylamide concentration layers. Before loading, an aliquot corresponding to 8 μg of Chl was taken from each sample, resuspended in 25BTH20G buffer [25 mM BisTris/HCl (pH 7.0), 20% (w/v) glycerol] to a final Chl concentration of 0.5 mg/ml and to a selected final n-dodecyl α-D-maltopyranoside (α-DDM) concentration. Second dimensions were performed in a Tricine-SDS PAGE system (Schägger, 2006). Gels after 2D were stained with the Serva Blue G Coomassie stain (SERVA Electrophoresis), subsequently, the gels were digitized with ImageQuant LAS4000 (GE, Healthcare). The data were preprocessed in ImageJ and analyzed with an R-project homemade script based on the workflow from Natale et al. (2011) with the modifications described in the section “Results.” The statistical analysis was performed using R-project and all the graphs were plotted using the ggplot2 package. After Coomassie staining the gels showed a fluctuating background drift, which necessitated a broad region of interest (ROI) selection and a constant background exclusion based on a threshold from local minima (see **Figure 2C**). To adjust for the variation in the staining/destaining and digitization steps during the gel processing (if not mentioned otherwise), a second normalization to total protein content (sum of the Integrated Optical Densities, IODs of all measured ROIs in a gel) was applied.

### Sucrose Gradients

Sucrose gradients were prepared according to Caffarri et al. (2009). Before loading on the sucrose gradient, thylakoid samples corresponding to 500 μg of Chl were solubilized in a final concentration of 0.6% α-DDM.

### Functional Antenna Size of PSII

To estimate the functional antenna size the measuring protocol was adapted from Dinç et al. (2012). The fluorescence induction curves (OJIPs) were measured with HandyPea (Hansatech) on dark acclimated (>1 h) intact leaves. A 1 s pulse of red light (650 nm) was given in the intensity range of 200–3500 μmol photons^∗^m^–2∗^s^–1^ (200, 300, 450, 600, 750, 900, 1200, 1500, 2000, 2500, 3000, and 3500 μmol photons^∗^m^–2∗^s^–1^). The leaf clips assured that the measurements for each light intensity were on the same spot of the first fully developed leaf, from 10 different plants. The dark acclimation periods between measurements of different light intensities were at least 10 min long. The fluorescence intensity is a function of the light intensity and to correct for this, measured fluorescence was normalized to the Photosynthetic Photon Flux Density (PPFD). Linear regression was performed to get the slope and the slope error of the in growth of the fluorescence intensity as a function of the light intensity at 300 μs.

## Results

### Long-Term Acclimation

#### General Plant Characterization

**FIGURE 1:**
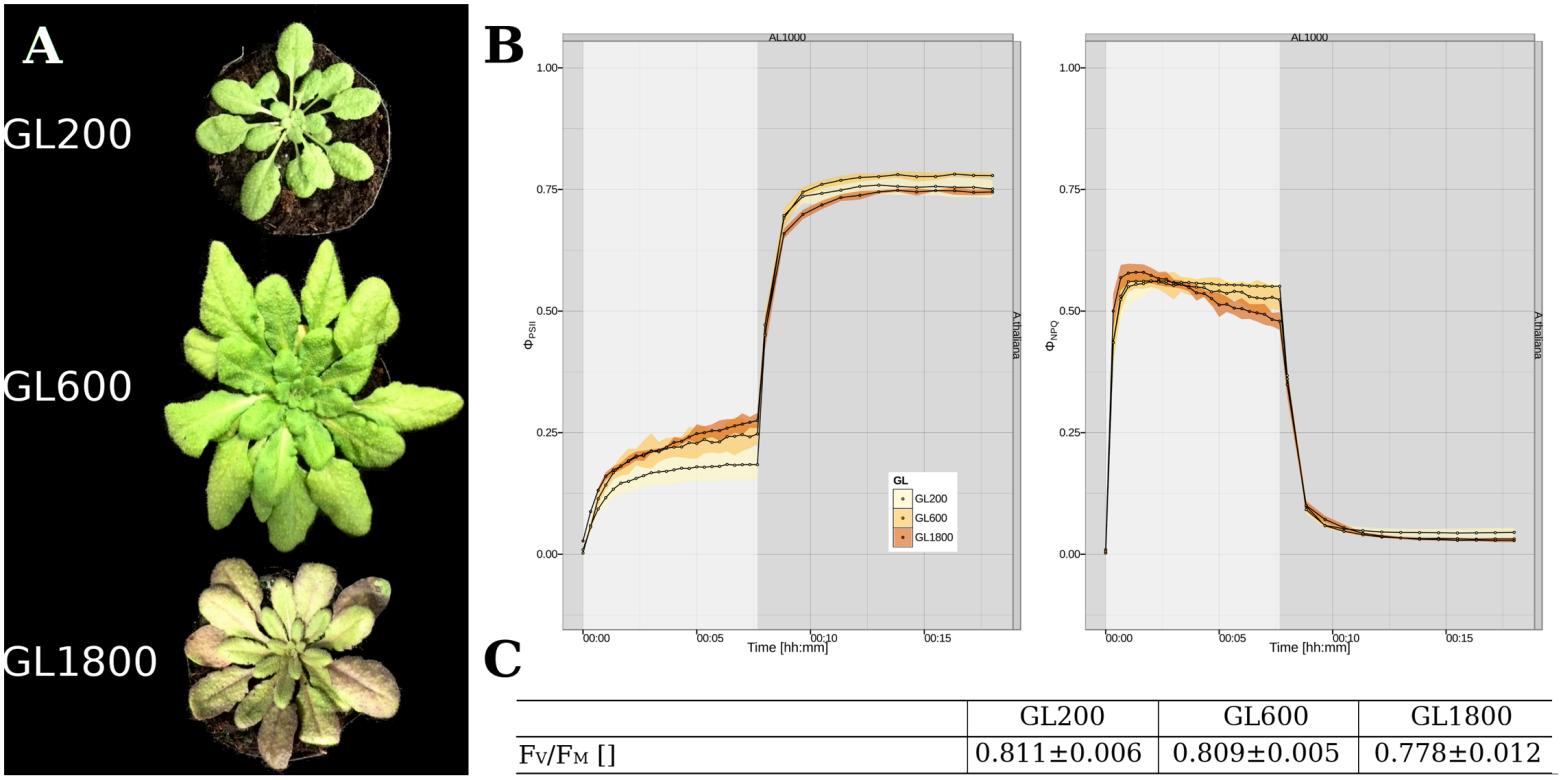
**General characterization of plants long-term acclimated to different light intensities. (A)** Visual appearance of the *Arabidopsis thaliana* plants grown under 200 (GL200), 600 (GL600), and 1800 μmol photons^∗^m^–2∗^s^–1^ (GL1800). **(B)** Parameters from the quenching analysis related with the energy distribution in PSII, Φ_PSII_ (Left panel) and Φ_NPQ_ (Right panel) during an 8 min illumination with 1000 μmol photons^∗^m^–2∗^s^–1^ and 10 min of darkness for plants grown under 200, 600, and 1800 μmol photons^∗^m^–2∗^s^–1^, respectively, as a yellow, orange and brown trace, (*n* = 4). The illumination and dark periods are marked, respectively, as gray and white background. **(C)** Dark fluorescence parameter *F*_V_/*F*_M_ of plants grown at all three light intensities (*n* = 4).

To investigate the long-term light acclimation of *A. thaliana*, plants were grown under three different light intensities: 200 (GL200), 600 (GL600), and 1800 μmol photons^∗^m^–2∗^s^–1^ (GL1800). As shown in **Figure 1A**, rosette and leaf morphology differed. Under GL200, plants had elongated petioles, the leaves were thin and had small oval leaf blades (Keller et al., 2011; Hersch et al., 2014). Under GL600, they grew faster, developed thicker leaves (Weston et al., 2000) with longer and broader leaf blades, and almost no petioles. Under GL1800, the rosettes were smaller, the older leaves brownish (Page et al., 2012), leathery thick, and the younger smaller, in larger number and concentrated around the central meristem.

The quenching analysis gives the possibility to observe time-related changes of energy partitioning between different pathways in PSII during light and dark acclimation (see **Figure 1B**). The parameter related with linear electron flow (LEF), Φ_PSII_, was higher when the plants were grown under higher light intensities (see **Figure 1B**, Left panel). As for the NPQ (see **Figure 1B**, Right panel), in steady state, Φ_NPQ_ was lower in plants grown in higher light intensities, but during the fast NPQ induction phase the HL grown plants were reaching the maximum faster than the other plants. The maximum efficiency of PSII (*F*_V_/*F*_M_) was also dependent on the light conditions and especially in HL, showed a lower value (see **Figure 1C**).

All these data show that our plants have the characteristics of plants acclimated to different light intensities covering a large range of light acclimation responses between shade-avoidance (GL200) and high-light acclimation responses (GL1800), with an intermediate light intensity (GL600).

#### Pigment Analysis

To characterize the range of changes in pigment composition, pigment analysis was performed after a night of darkness (see **Table 1**). In plants grown under GL1800 the Chl content on a fresh leaf weight basis was almost half that under GL200. The Chl *a*/*b* ratio increased under higher light intensities, in agreement with a reduction of the antenna size (Andersson, 1996; Ballottari et al., 2007; Kouřil et al., 2013). The chlorophyll/carotenoid (Chl/Car) ratio decreased in plants grown at higher light intensities. As for the carotenoid composition, increased levels were observed for all of them under HL. In the thylakoids, Zeaxanthin (Zea) was observed in very small amounts in plants grown under GL200 and increased in plants grown at higher light intensities, indicating that Zea is not completely re-converted to violaxanthin in darkness.

**Table 1:** Pigment analysis of plants grown under different light conditions.

	GL200	GL600	GL1800
Chls/fresh weight [mg/g]	0.8621 ± 0.1156	0.7821 ± 0.0354	0.3893 ± 0.0986
Chl a/b	3.235 ± 0.022	3.249 ± 0.006	3.562 ± 0.047
Chl/Car	3.905 ± 0.024	3.698 ± 0.015	3.228 ± 0.054
Neo/100 Chls	3.424 ± 0.048	3.743 ± 0.031	4.004 ± 0.178
Vio/100 Chls	2.753 ± 0.033	3.035 ± 0.005	4.106 ± 0.183
Ant/100 Chls	0.196 ± 0.023	0.381 ± 0.010	0.621 ± 0.030
Lut/100 Chls	12.364 ± 0.0125	12.900 ± 0.041	13.981 ± 0.284
Zea/100 Chls	0.195 ± 0.005	0.296 ± 0.027	0.678 ± 0.018
β-Car/100 Chls	6.677 ± 0.017	6.688 ± 0.069	7.592 ± 0.176
Cars/100 Chls	25.609 ± 0.157	27.044 ± 0.108	30.98 ± 0.513
(Z + 0.5 × A)/(Z + A + V)	0.0932 ± 0.002	0.131 ± 0.007	0.183 ± 0.003

*Total chlorophylls (Chls) were quantified by fitting the 80% acetone extracts from a leaf from five different plants (n = 5) grown under GL200, 600, and 1800 and normalized to the fresh weight. The chlorophyll a/b (Chl a/b) ratio and chlorophyll/carotenoid (Chl/Car) ratio was determined in the same way from isolated thylakoid membranes in three repetitions (n = 3). The same extracts were used for the quantification by HPLC of the carotenoids: neoxanthin (Neo), violaxanthin (Vio), anteraxanthin (Ant), zeaxanthin (Zea), lutein (Lut) and* β-*carotene (β-car). All carotenoids were calculated per 100 Chls.*

#### Structural Antenna Size

**FIGURE 2:**
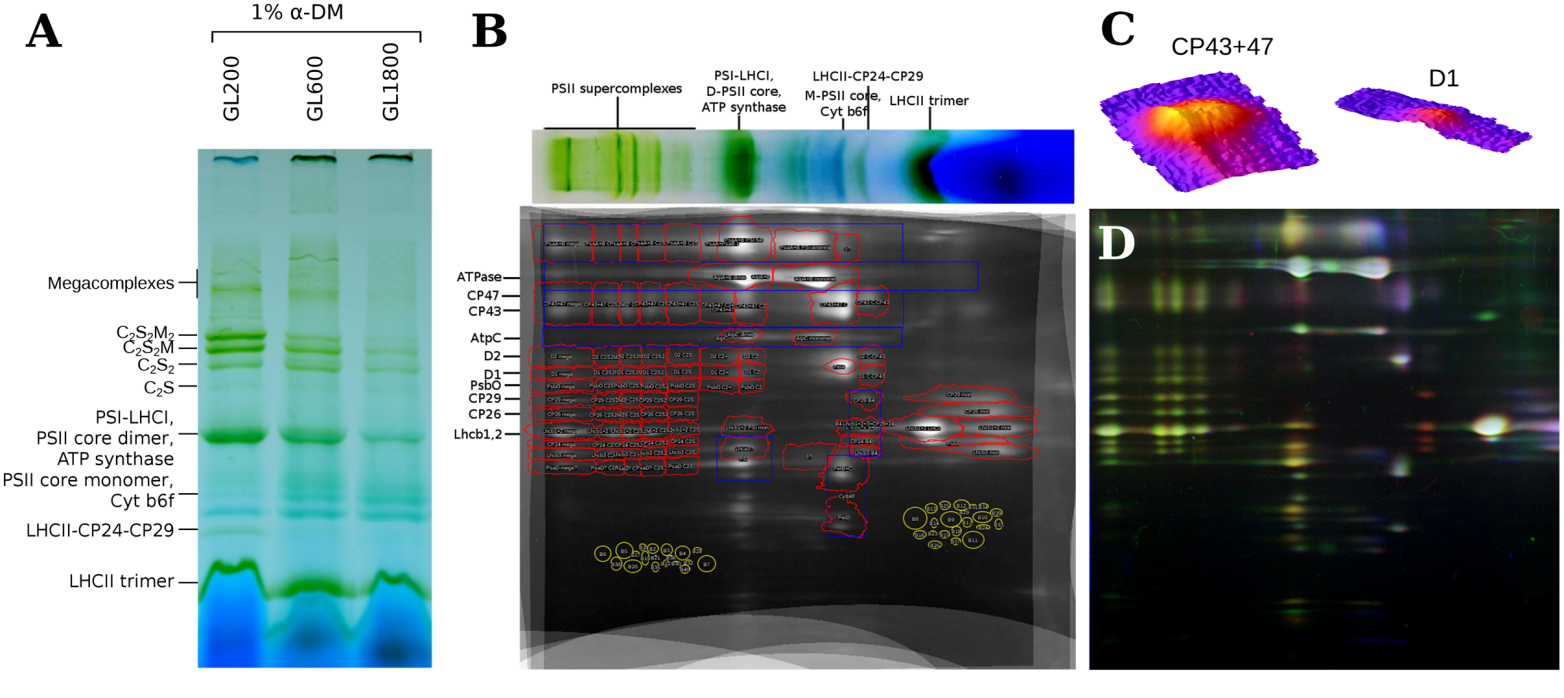
**2D-PAGE of thylakoids isolated from plants grown under different light intensities. (A)** The BN-PAGE of thylakoids isolated from plants grown under GL200, 600, and 1800, solubilized with 1% α-DDM. **(B)** Sum of nine 2D-PAGE gels of isolated thylakoids from plants grown under all light conditions. The red contours represent ROIs of selected proteins in specific complexes. The blue contours represent the integrated areas for quantification of a selected protein independent of the complex. On the top, a lane from the BN-PAGE is shown. **(C)** Overlap of the gels of thylakoids from plants grown under GL200, 600, and 1800, that are shown in the red, green, and blue channels, respectively. **(D)** 3D surface plots of CP43 + 47 and D1 megacomplex ROIs, respectively.

To determine the composition of the thylakoid membranes, 2D-PAGE was performed. The isolated thylakoid membranes were solubilized with α-DDM (1% final concentration), a mild detergent that preserves the PSII supercomplexes (see **Figure 2A**). In the second dimension, the proteins of which these complexes are composed, were separated in a denaturing gel (Tricine-SDS PAGE; see **Figure 2B**) allowing a relative quantification of the proteins in each complex. To test and ensure the reproducibility of the results, three repetitions per condition were performed. A workflow from Natale et al. (2011) was adapted to perform a half-automated, qualitative and quantitative analysis of the most abundant thylakoid membrane proteins. After the warping step, the alignment of the gels was accurate enough for a qualitative analysis (see **Figure 2D**). The dot patterns on the 2D-gel were the same for plants grown under all light conditions, which suggests that there are no qualitative differences in the protein composition of the most abundant thylakoid proteins. The identification of specific proteins on 2D-gels was performed based on previous work (Aro et al., 2005; Andaluz et al., 2006; Caffarri et al., 2009; Takabayashi et al., 2013).

To automate the quantification of proteins from the 2D-PAGE gels a ROI map (see **Figure 2B**) was created based on an averaged image of nine, warped 2D gels. For the quantification of the (super)complexes, multiple proteins representative of each complex were selected.

**FIGURE 3:**
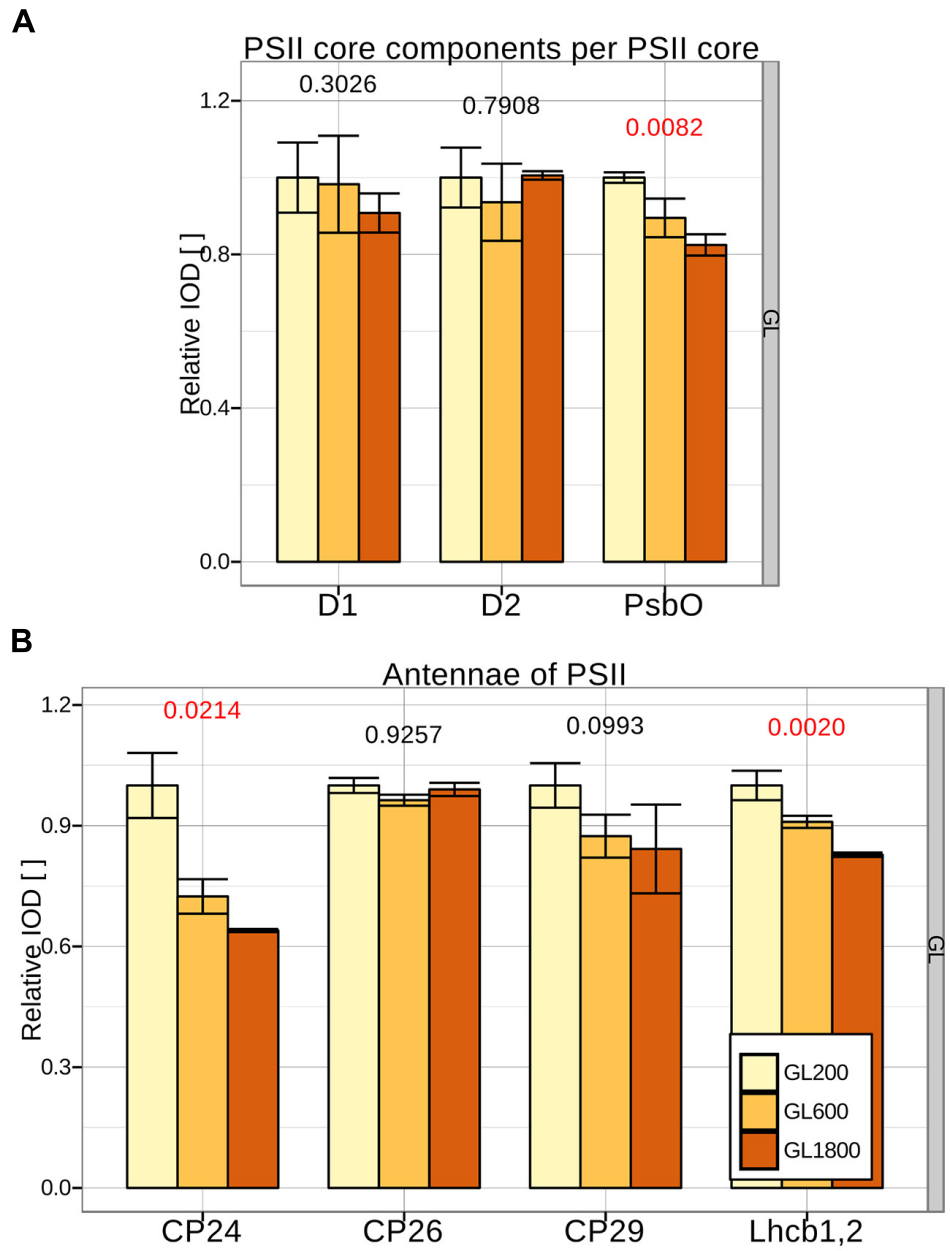
**Changes in the PSII composition during long-term acclimation to different light intensities. (A)** The amount of PSII core proteins: D1, D2, and PsbO per CP43, 47 was quantified in isolated thylakoids from plants grown under GL200, 600, and 1800 (respectively, yellow, orange, and brown fill) separated during 2D-PAGE in three different repetitions (*n* = 3). **(B)** The amount of minor PSII antennae: CP24, CP26, CP29, and Lhcb1,2 per PSII core was quantified in isolated thylakoids from the same samples. Numbers below the bars are the *p*-values of ANOVA’s *F*-test on a specific group. Red color signifies rejection of the null hypothesis that the response to all growth lights is the same (α < 0.05), black its acceptance.

To determine if changes in the PSII antenna size occur, antenna proteins have to be quantified relative to a PSII core protein that is present in each PSII complex. Candidates were: D1, D2, CP43, and CP47. The dots on the gel of CP43 and CP47 were more pronounced than the dots of D1 and D2 (see **Figure 2C**). Since the small MW-difference of CP43 and CP47 led to an incomplete separation, the averaged IOD of the CP43 and CP47 dots was chosen (besides the C-CP43 fraction where only CP47 is present). This reference was shown to follow changes in D1 and D2 closely, under all light conditions (see **Figure 3A**), confirming that it accurately reflected the amount of PSII core. The small differences in stoichiometry between conditions and the quite pronounced standard deviation in **Figure 3** were due to the low signal from D1 and D2. Looking at the other components of the PSII core, we observed, for plants grown under higher light intensities, a decrease in the amount of PsbO per core, which could be due to the dissociation of PsbO during photoinactivation of PSII complexes (Hundal et al., 1990).

#### Antennae of PSII

The minor antennae CP24, CP26, and CP29 and Lhcb1,2 (major components of LHCII trimers) were quantified from the sum of the corresponding dots from all PSII complexes and normalized to the amount of the PSII core (see **Figure 3B**). Because of the sequence specific affinity of Coomassie for the proteins, a second normalization was performed using as reference the plants grown under GL200. When the plants grew under higher light intensities, the amount of CP24 decreased, whereas the amounts of CP26 and CP29 were maintained at a similar level, in agreement with previous results (Ballottari et al., 2007; Kouřil et al., 2013). Under GL1800, the amount of LHCII per PSII decreased to ∼80% of the value observed under GL200. Note that at GL200 the antenna size of *A. thaliana* is already reduced compared to the values observed when lower light intensities were used: 100 μmol photons^∗^m^–2∗^s^–1^ in Ballottari et al. (2007) and Kouřil et al. (2013).

#### PSII Antenna Size Heterogeneity

**FIGURE 4:**
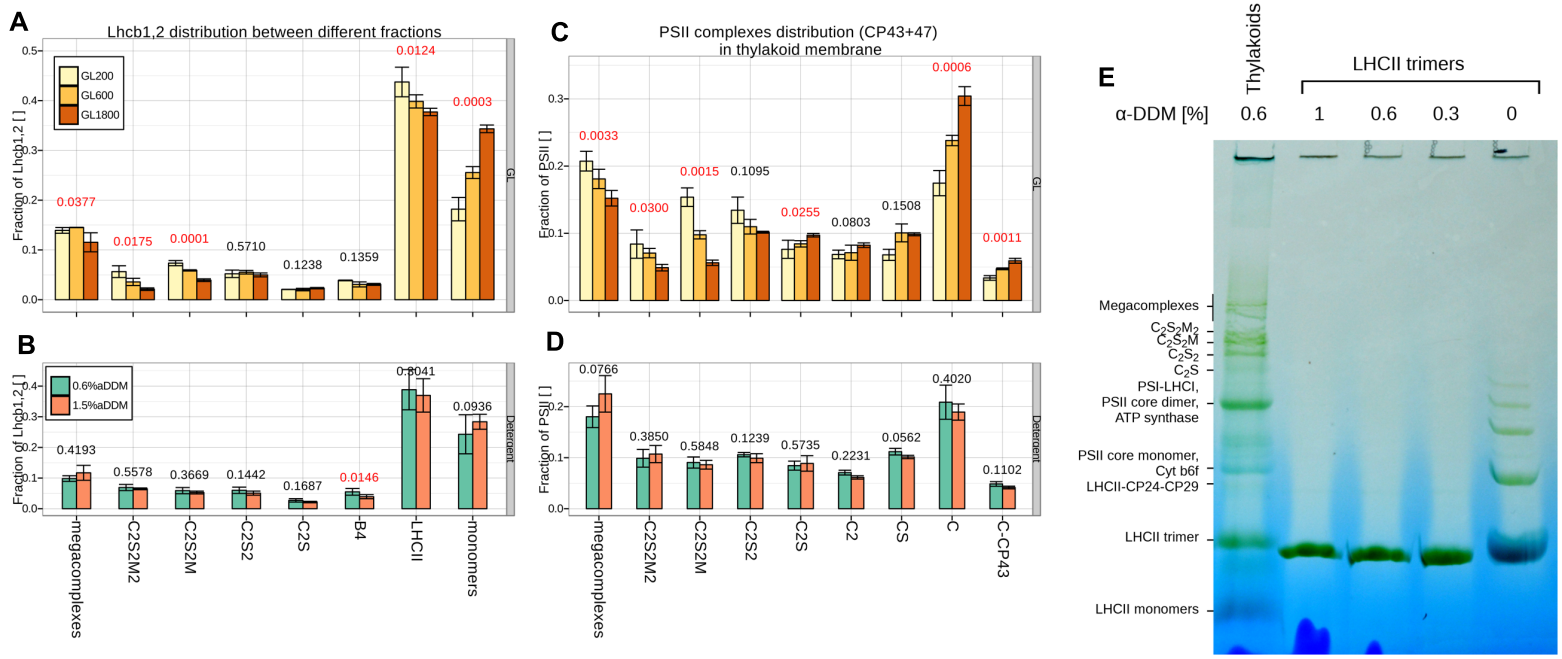
**Growth light (GL) and detergent influence on PSII complexes and Lhcb1, 2 distribution.** The distribution of PSII between different PSII complexes according to the Lhcb1,2 **(A,B)**: supercomplexes (megacomplexes, C_2_S_2_M_2_, C_2_S_2_M, C_2_S_2_, C_2_S), different complexes (CP29 + LHCII + CP24 complex, B4; Light Harvesting complex of PSII, LHCII) and monomers or CP43 and CP47 **(C,D)**: supercomplexes (megacomplexes, C_2_S_2_M_2_, C_2_S_2_M, C_2_S_2_, C_2_S, CS) or different core complexes (core dimers, C_2;_ core monomers, C; core monomers without CP43, C-CP43) in isolated thylakoids separated during 2D-PAGE and quantified from three different gels (*n* = 3). Thylakoids were taken from plants grown under GL200, 600, and 1800 (respectively, yellow, orange, and brown fill; **A,C** panels) or only GL200, but were solubilized in 0.6 and 1.5% α-DDM (respectively, green and blue cyan fill; **B,D** panels). Numbers above the bars are the *p*-values of ANOVA’s *F*-test on a specific group. Red color signifies rejection of the null hypothesis that the response to all GLs is the same (α < 0.05), black its acceptance. **(E)** BN-PAGE of thylakoids solubilized in 0.6% α-DDM and LHCII trimers (3 μg of Chl) solubilized in 0, 0.3, 0.6, and 1% of α-DDM.

Previous work has indicated that the differences in antenna size, observed upon light acclimation, lead to changes not only in the amount of “extra” LHCII but also in the relative amount of the PSII (super)complexes (Wientjes et al., 2013a). To quantify these changes we estimated the core protein distribution of PSII in PSII supercomplexes (megacomplexes, C_2_S_2_M_2_, C_2_S_2_M, C_2_S_2_, C_2_S, CS) and core complexes (core dimers, C_2_; core monomers C; core monomers without CP43, C-CP43). The amount of CP43 and CP47 was normalized to the total amount of these proteins for each condition (see **Figure 4C**). When the plants were grown under GL200, PSII was observed mostly in the form of megacomplexes, C_2_S_2_M, C_2_S_2_ and core monomers. At higher light intensities, the fraction of large supercomplexes decreased (from megacomplexes to C_2_S_2_M), while the amount of smaller complexes increased (C_2_S, C, C-CP43).

A similar trend was observable when looking at Lhcb1,2 distributed between the different fractions (see **Figure 4A**). Under all conditions the trimeric fraction was the most abundant containing 35–45% of the LHCII pool. The monomeric fraction was, however, also large, representing approximately 20% of the LHCII population in GL200 and increasing to 35% in HL. The rest of LHCII was associated with the supercomplexes. During acclimation to higher light intensities, there was a relative decrease in the amount of Lhcb1,2 associated with the large PSII supercomplexes (C_2_S_2_M_2_, C_2_S_2_M). Accumulation of core proteins did not correlate with the accumulation of LHCII trimers, but with the increase of Lhcb1,2 monomers.

To rule out the possibility of solubilization artifacts, the results were validated by performing the same 2D-PAGE analysis on thylakoids from the plants grown under GL200, solubilized with 0.6 and 1.5% of α-DDM (see **Figures 4B,D**). The results show that an almost threefold increase in detergent to protein ratio did not influence the core distribution of PSII (see **Figure 4D**). Similarly, in the case of the antenna, only the amount of LHCII-CP24-CP29 (B4) slightly decreased when using a high detergent concentration, while no changes were observed for the other complexes (see **Figure 4B**).

To further verify if the presence of LHCII monomers could be the result of solubilization (see **Figure 4E**), purified LHCII trimers were directly solubilized with different detergent concentrations and loaded on a BN-PAGE. No monomerization was observed under any of the solubilization conditions, confirming the high stability of the trimers to detergent treatment. It is interesting to observe that LHCII trimers loaded on the BN gel without the addition of detergent (**Figure 4E**, most right lane) form dimers, trimers, tetramers, and higher assemblies, indicating that complexes can aggregate in the gel, contrary to what was previously assumed (Ilioaia et al., 2008).

#### Functional Antenna Size of PSII

**FIGURE 5:**
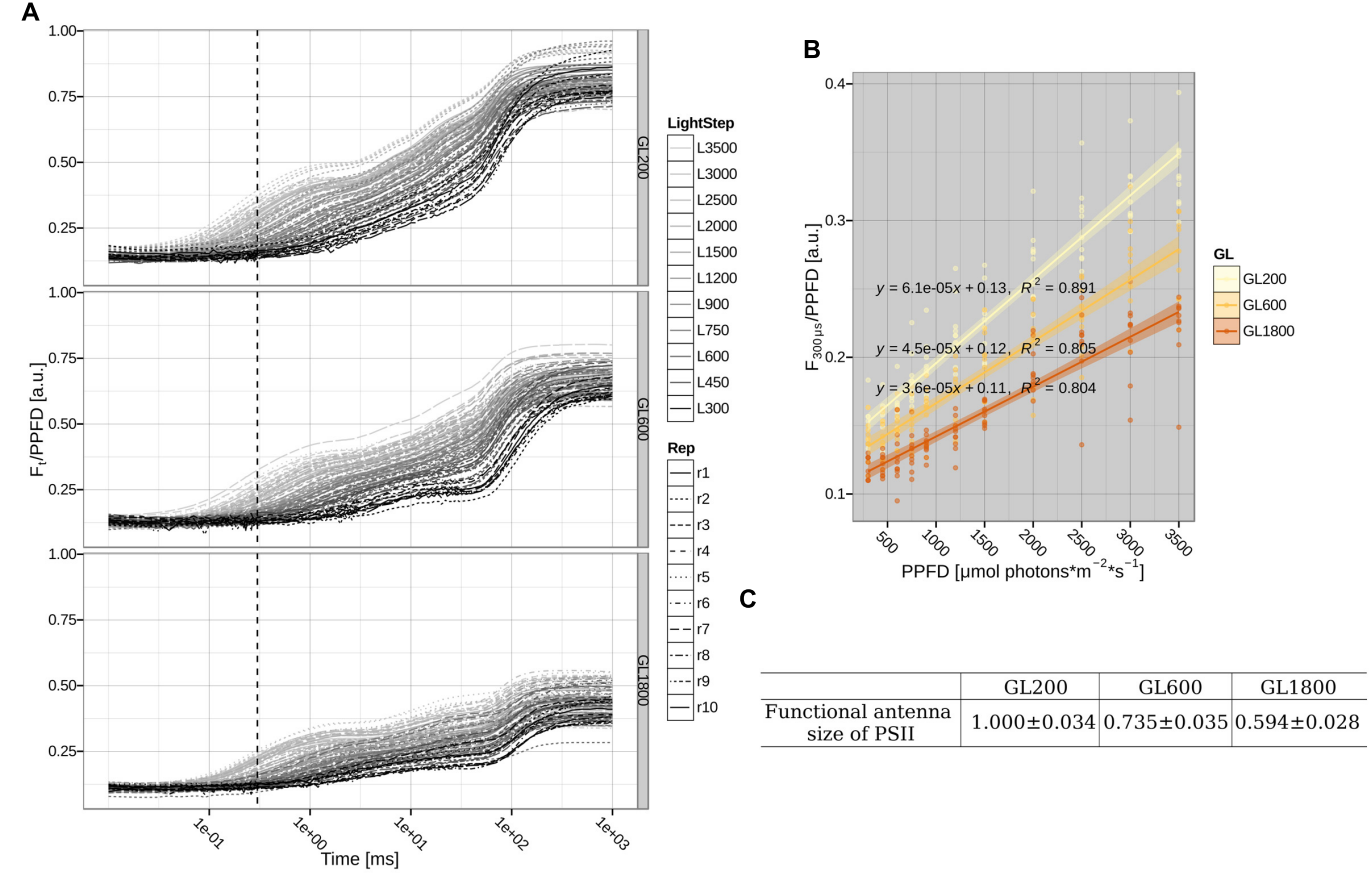
**Functional antenna size of PSII. (A)** Direct measurements of chlorophyll *a* fluorescence *in folio* during a 1 s pulse of 300, 450, 600, 750, 900, 1200, 1500, 2000, 2500, 3000, and 3500 μmol photons^∗^m^–2∗^s^–1^ were normalized to the photosynthetic photon flux density (PPFD). The dotted, vertical line shows the 300 μs time point. Shown traces for 10 different plants (Rep1-10, respectively, represented by different line types) grown under GL200, 600, and 1800 (respectively, Top, Central, and Bottom Left panels). **(B)** Relationship between normalized fluorescence at 300 μs and PPFD fitted with a linear regression. The fitted lines with their standard error are shown as lines with shadows. Individual data points are from the plants grown under GL200, 600, and 1800, yellow, orange, and brown color, respectively. **(C)** The slope and standard error of a fit of the normalized fluorescence at 300 μs against PPFD relationship corresponding to the functional antenna size of PSII on 10 different plants (*n* = 10) grown under GL200, 600, and 1800.

In the next step, we determined the changes in the functional antenna size (the antenna that is able to transfer the absorbed energy to the PSII Reaction Center, RC), by measuring the fluorescence rise, at different light intensities, on leaves (see **Figure 5A**). The slope of the normalized fluorescence at 300 μs vs. the light intensity (see **Figure 5B**) is proportional to the absorption cross-section of PSII and is then used to determine the functional antenna size of PSII (Dinç et al., 2012). The data show that in plants grown under GL600 and GL1800, the functional antenna size dropped to 73 and 59%, respectively, of the value of plants grown under GL200 (see **Figure 5C**).

**FIGURE 6:**
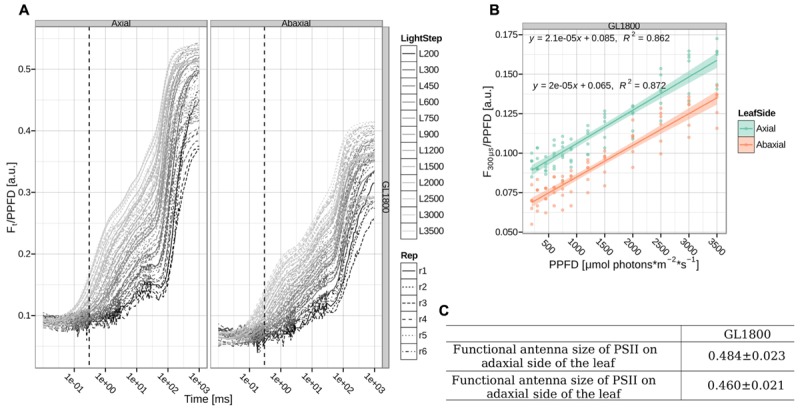
**Functional antenna size of PSII. (A)** Direct measurements of chlorophyll *a* fluorescence *in folio* during a 1 s pulse in the range of 200–3500 μmol photons^∗^m^–2∗^s^–1^ were normalized to the PPFD. The dotted, vertical line shows the 300 μs time point. Shown traces for six different plants (Rep1-6, respectively, represented by different line types) grown under GL1800 and measured on the axial or abaxial side of the leaf (respectively, Left and Right panel). **(B)** Normalized fluorescence at 300 μs as a function of PPFD on plants from GL1800 measured on axial and abaxial side of the leaf (cyan and orange points, respectively). The linear regression fit and its error are shown as a line with a shadow. **(C)** The relative slope and standard error of a fit of the normalized fluorescence at 300 μs against PPFD relationship corresponding to the functional antenna size of PSII on six different plants (*n* = 6) grown under GL1800. Data normalized to GL200 measurements performed on adaxial side of the leaves.

The discrepancy between the functional and structural antenna size measurements may be caused by several factors. The protein quantification was performed on thylakoid membranes isolated from the whole leaf and represents thus an averaged population. This can be important because during growth a light gradient within a leaf, with cell layers deeper in the leaf being exposed to lower light intensities, leads to a range of differently acclimated chloroplasts. In the top layers PSII can have smaller antennae than in the bottom layer (Nishio et al., 1993; Vogelmann and Han, 2000; Evans and Vogelmann, 2003). For the functional measurements, fluorescence is emitted in response to excitation with red light, which is absorbed strongly by the top cell layers causing a strong light gradient inside the leaf and the measured fluorescence emission is derived mainly from chloroplasts in the top cell layers (Terashima et al., 2009). To check if this is the case here as well, we measured the PSII functional antenna size from the axial and abaxial sides of the leaves of plants grown under GL1800 (see **Figure 6**), where the leaves are thickest and the anticipated effect should be most pronounced. We did not observe any significant differences in the antenna sizes measurements between the axial and abaxial side. The smaller antenna size observed in this experiment, when compared to the previous batch (see **Figures 5** and **6**) should be ascribed to the biological variation between the rounds of growth, as the standard errors within each set were small. This leads to the conclusion that the difference between functional and structural measurements did not originate from the shallow probing during fluorescence measurements. A series of other effects can influence the measurements of the functional antenna size, including changes in the leaf structure, as well as chloroplast and membrane organization. However, the large difference between the protein content and the functional measurements suggests also that in HL, part of the antenna does not transfer the absorbed light efficiently to the RC.

### Short-Term Light Stress

Next, we studied the effect of short light stress on the PSII super- and sub-complexes and the possible transition between short- and long-term strategies by following the first 6 h of HL treatment. The thylakoid membranes were isolated from plants grown under GL200 and transferred for 0.5, 2 and 6 h to GL1800. All plants analyzed in this experiment were light adapted and following the treatment the leaves were immediately cooled in an ice/water mixture. To validate if the short-term response was induced properly, pigment analysis was performed (see **Table 2**). The deepoxidation level of xanthophylls increased sharply to around 40% during the first 30 min, and as plants were kept longer under GL1800 the level reached 55% after 6 h.

**Table 2:** Pigment analysis during the first 6 h of HL.

	0 h	0.5 h	2 h	6 h
Chl a/b	3.210 ± 0.014	3.231 ± 0.005	3.240 ± 0.020	3.241 ± 0.006
Chl/Car	4.193 ± 0.007	4.036 ± 0.019	3.981 ± 0.017	3.740 ± 0.013
Neo/100 Chls	3.269 ± 0.139	3.440 ± 0.095	3.416 ± 0.080	3.613 ± 0.193
Vio/100 Chls	3.157 ± 0.181	1.930 ± 0.067	1.804 ± 0.081	1.822 ± 0.101
Ant/100 Chls	0.416 ± 0.015	0.634 ± 0.021	0.725 ± 0.017	1.001 ± 0.042
Lut/100 Chls	14.989 ± 0.654	15.921 ± 0.257	15.772 ± 0.338	16.218 ± 0.643
Zea/100 Chls	0.274 ± 0.015	1.129 ± 0.053	1.599 ± 0.154	2.296 ± 0.129
β-Car/100 Chls	1.739 ± 0.939	1.720 ± 0.372	1.797 ± 0.619	1.781 ± 1.125
Cars/100 Chls	23.846 ± 0.041	24.776 ± 0.118	25.116 ± 0.109	26.734 ± 0.096
(Z + 0.5 × A)/(Z + A + V)	0.125 ± 0.003	0.391 ± 0.004	0.474 ± 0.010	0.546 ± 0.002

*Pigments were extracted from thylakoids from plants grown under GL200 and transferred for 0.5, 2 and 6 h. The chlorophyll a/b (Chl a/b) ratio and chlorophyll/carotenoid (Chl/Car) ratio was determined the same way from isolated thylakoid membranes in three different repetitions (n = 3). The same extracts were used for the quantification by HPLC of the carotenoids: neoxanthin (Neo), violaxanthin (Vio), anteraxanthin (Ant), zeaxanthin (Zea), lutein (Lut) and* β-*carotene (β-car). All carotenoids were calculated per 100 Chls.*

**FIGURE 7:**
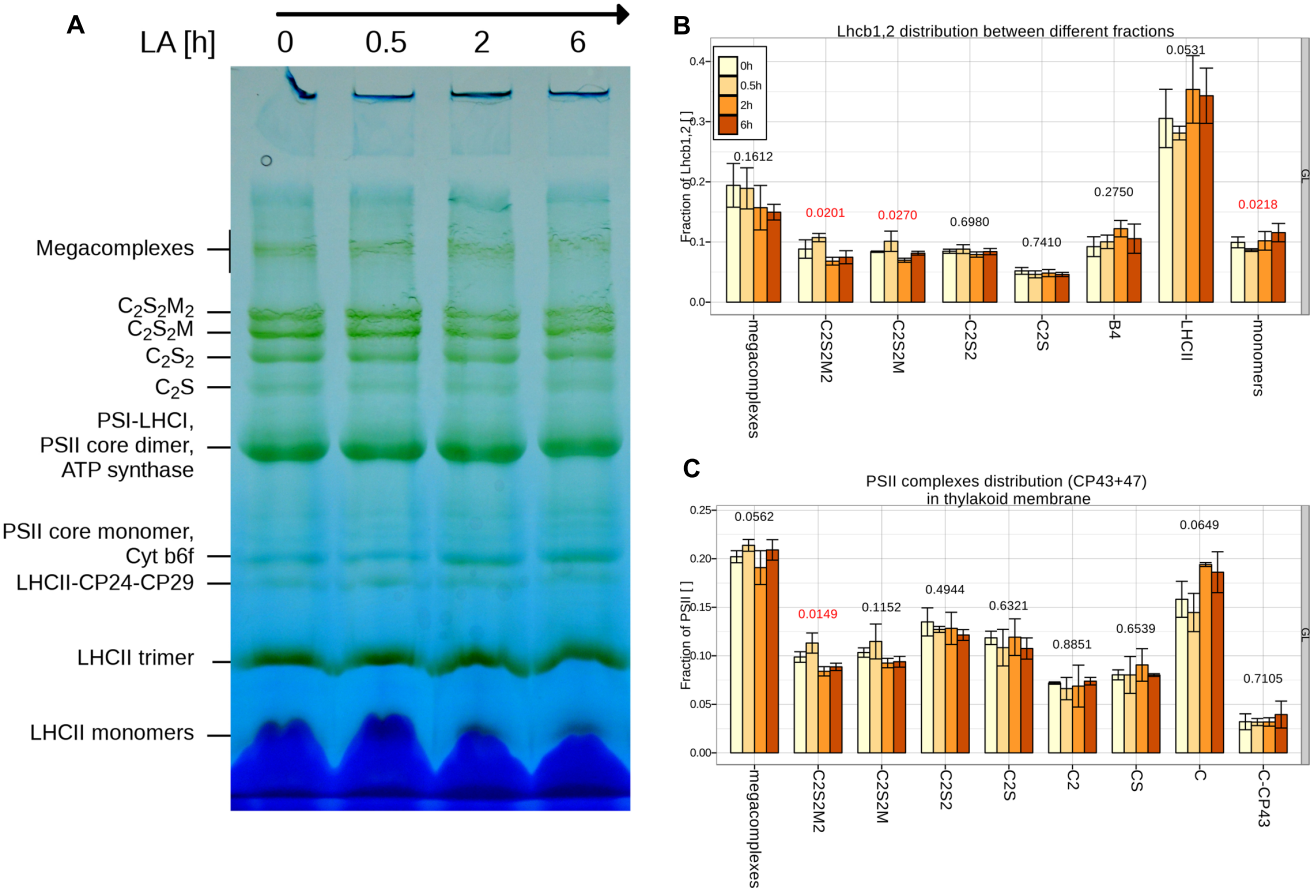
**Changes in the distribution of PSII complexes during the first 6 h of HL. (A)** BN-PAGE of thylakoids from plants grown under GL200 and transferred for 0.5, 2 and 6 h. **(B)** The distribution of PSII between different PSII complexes according to Lhcb1,2: supercomplexes (megacomplexes, C_2_S_2_M_2_, C_2_S_2_M, C_2_S_2_, C_2_S), different complexes (CP29 + LHCII + CP24 complex, B4; Light Harvesting complex of PSII, LHCII) and monomers; or CP43 + 47 **(C)**: core dimers, C_2_; core monomers, C; core monomers without CP43, C-CP43 in isolated thylakoids separated during 2D-PAGE and quantified from three different gels (*n* = 3). Thylakoids were obtained from plants grown under GL200 and transferred for 0.5, 2 and 6 h to GL1800. Red color signifies rejection of the null hypothesis that the response to all growth light is the same (α < 0.05), black its acceptance.

2D-PAGE analysis was used to determine possible changes in the PSII organization and composition (see **Figure 7**). No changes in the distribution of complexes and supercomplexes were observed upon 30 min of HL. A decrease in the mega and supercomplexes containing trimer M was observed after 2 h of treatment, accompanied by a small relative increase of LHCII-CP24-CP29 and LHCII trimers. The amount of LHCII monomers increased only slightly during the treatment.

## Discussion

Differences in the growth light intensity have been shown to lead to changes in the protein composition and in the organization of the thylakoid membrane of plants. Long-term acclimation is known to reduce the number of LHCII (and CP24) subunits present in the membrane (Ballottari et al., 2007; Kouřil et al., 2013), while short-term response is suggested to consist of a reorganization of the membrane with the disconnection of part of the antenna from PSII (Betterle et al., 2009; Holzwarth et al., 2009; Johnson et al., 2011). Both these strategies should then result in a change in the antenna size of PSII. In this work, we have investigated this aspect systematically by studying how short-term light responses and long-term acclimation affect the quaternary structure of PSII super- and sub-complexes.

### *In Vitro* vs. *In Folio*

Native gels are a powerful tool to study the presence and the different aggregation states of photosynthetic complexes and supercomplexes (Aro et al., 2005; Andaluz et al., 2006; Danielsson et al., 2006; Järvi et al., 2011). However, there are several issues that may complicate the extrapolation of the biochemical data to *in folio* systems and we had started our investigation by checking the validity of the analysis and answering several questions. Do the differences observed upon solubilization of the membranes reflect the situation *in folio*? Are the results biased by the solubilization steps? To minimize the solubilization artifacts we used low concentrations of a mild detergent (but strong enough to solubilize both grana and lamellae). We also used different amounts of detergent in a range that covers the possible differences in protein/lipids/detergent ratios in the various samples. The results show no influence of the detergent concentration on the PSII supercomplex distribution. Despite the fact that these results indicate that the observed differences are not due to the solubilization, we should keep in mind that the purification procedure has likely an effect on very labile complexes and our analysis gives the lowest threshold for the largest fractions of PSII supercomplexes.

The other essential question is how to analyze the gel and check the reproducibility of the results. To achieve this goal we adapted for our purposes the workflow from Natale et al. (2011). We used only open source or homemade programs based on ImageJ or R. To correct for differences in acrylamide polymerization and electrophoresis we had to warp the gels in ImageJ (UnwarpJ package). To correct for the differences in Coomassie staining between gels (fluctuating background drift) we performed a local normalization to the minimum of a broad ROI. The selectivity of the background exclusion was tested on empty parts of the gel (data not shown) giving satisfactory results. The sample loading (protein content) on a BN-PAGE is often slightly different, especially, if working with plants grown under non-standard conditions, as some pellet is present after solubilization. In this respect it is important to mention that the pellet of samples from plants grown under different conditions has a composition similar to that of the solubilized fraction (data not shown) excluding the possibility that there was selective solubilization. Additionally, when needed, we performed a normalization to total protein content (we had around 97 recognized protein dots) or to a reference protein. It is crucial to remember that the absolute values are difficult to obtain as the binding mechanism of Coomassie is complex (Georgiou et al., 2008). Additionally, we observed an influence of freezing and thawing on the quality of the samples, so each sample was run fresh after isolation or after only one freeze-thaw cycle. It should be kept in mind that all data obtained upon freezing and thawing the membranes multiple times are unreliable.

### Acclimation of PSII (Super)Complexes

Upon HL acclimation a reduction in the relative amount of PSII mega and supercomplexes containing trimer M at increasing light intensities was observed. This was expected on the basis of the reduction of CP24 and Lhcb3 (Ballottari et al., 2007; Kouřil et al., 2013), which are essential for the stabilization of trimer M (Kovács et al., 2006; Caffarri et al., 2009). Under the same conditions, the relative amount of core monomers significantly increased. This is in agreement with the idea that the source of the PSII core monomers is the constant activity of the repair cycle, which is higher in HL (Aro et al., 2005).

No significant changes in any of the PSII supercomplexes could be observed during the first 30 min, when the amount of zeaxanthin is already high, while a relative decrease in the supercomplexes containing trimer M started to be observed only after 2 h of HL exposure. Although we cannot exclude that during the fast phase of NPQ the antenna is disconnected and then it reconnects again during the preparation, we can conclude that the presence of zeaxanthin does not have an effect on the organization of the supercomplexes and that the long term acclimation is already active after 2 h of HL.

### LHCII Monomers vs. Trimers

It is interesting to see that no changes are observed in the ratio between LHCII trimers and monomers in the first hours of HL, while an increase in LHCII monomers is visible upon long-term acclimation to HL. The data suggest that a population of LHCII monomers exists in the membrane as the high stability of LHCII trimers to detergent treatment seems to exclude that their presence is an artifact of the purification. It has been proposed that the LHCII monomer can be partially responsible for the irreversible part of the quenching (Garab et al., 2002). More recently it was shown that LHCII in the membrane of the green alga *Chlamydomonas reinhardtii* exists in different quenching states and that the ratio between these states depends on the growth light conditions (Tian et al., 2015). Here, we observed that the PSII maximum efficiency is lower in HL than in LL indicating that a larger part of the absorbed energy is not used for photochemistry. Moreover, a large difference between the antenna size at the protein and at the functional level is observed in HL. It is thus tempting to speculate that LHCII monomers observed in high amount in HL are less efficient in transferring energy to the RC and act as a reservoir of LHCII.

## Author Contributions

RC conceived the research. LB, GS, and RC designed the research. LB, performed most of the experimental work. GS performed the antenna size measurements. LB and RC wrote the paper. All the authors contributed to the final version.

## Conflict of Interest Statement

The authors declare that the research was conducted in the absence of any commercial or financial relationships that could be construed as a potential conflict of interest.
